# Experiences, learnings and perspectives in the regulation of agricultural biotechnology: the view from Argentina

**DOI:** 10.3389/fbioe.2025.1600642

**Published:** 2025-06-06

**Authors:** Dalia M. Lewi, Perla Godoy, Facundo Simeone

**Affiliations:** ^1^ Dirección Nacional de Vinculación Tecnológica, Instituto Nacional de Tecnología Agropecuaria, Buenos Aires, Argentina; ^2^ Coordinación de Innovación y Biotecnología, Secretaría de Agricultura, Ganadería y Pesca, Buenos Aires, Argentina; ^3^ Dirección Nacional de Bioeconomía, Secretaría de Agricultura, Ganadería y Pesca, Buenos Aires, Argentina

**Keywords:** agricultural biotechnology, regulatory diplomacy, biosafety, regulation, biodevelopment

## Abstract

Argentina has established itself as global leader in setting enabling regulations for agricultural biotechnology. Between 2020 and 2023 the responsible administration strengthened this position through the adoption of innovative regulations for New Breeding Techniques and fostering broad international collaboration. The experience accumulated during this period serves to illustrate best practices, current shortcomings, anticipate future challenges, and point to the solutions that the sector will need in the next few years.

## Introduction

Modern agricultural biotechnology is the technological application of genetic engineering tools to improve crops, microorganisms, and livestock. Its primary objective is to provide benefits to farmers, consumers, industry, human and animal health, and the environment. This technology aims to increase agricultural production, reduce production costs, make more efficient use of resources, promote resilience to climate change while preserving agro-ecosystems, and increase the safety and quality of food.

In 1991, stakeholders within the agricultural sector approached the Argentine government expressing their interest in conducting experimental activities involving genetically modified (GM) plants and seeds. In response, the administration promptly created the National Advisory Commission on Agricultural Biotechnology (CONABIA for its acronym in Spanish). At the time, Argentina already employed professionals with a comprehensive scientific and technical background in modern agricultural biotechnology and was well equipped to fulfil this task. This capacity was recognised recognized by the Food and Agriculture Organization of the United Nations (FAO) in 2014, when CONABIA was nominated as the centre of reference for agricultural biosafety, a recognition still held to date^1^. CONABIA advises decision-makers on the authorization of activities related to genetically modified organisms (GMOs) for agricultural use, whether animals, plants or microorganisms, both for experimental activities and for the commercialization thereof. CONABIA professionals are highly qualified and keep up with the latest scientific advances on modern biotechnology techniques, as demonstrated by the pioneering work performed on the regulation of new breeding techniques, where Argentina was the first country to establish a framework for the assessment of these products.

### Actions and public policy proposals

Over the years, different Argentine administrations have been adding depth and innovation to regulatory management, in line with the latest scientific advances. In 2020, the National Directorate of Bioeconomy, which encompassed both the Biotechnology sector and CONABIA, proposed six guiding principles to develop actions and public policy proposals (see Table 1). The proposed principles were not only focused on generating capacities to strengthen regulatory areas. They also focused on facilitating innovation, promoting local research that applied science and technology to develop value-added products for the country. Unlike other countries, where regulatory systems for emerging technologies-such as gene editing-are not yet established or still require substantial capacity building to address regulatory challenges Argentina addressed these challenges over a decade ago. This was made possible by investing in highly trained scientific and technical work force.

**TABLE 1:** Guiding principles for Biotechnology public policy proposals.

i)	Support local bio-developments in biotechnology, bio-inputs, biomaterials, and bioenergy
ii)	sustain and improve the regulatory system, improve regulations and adapt them to local bio-developments
iii)	promote developments with added value at the source
iv)	contribute to the development of the bioeconomy and its application to the country’s agriculture, based on the development of bio-inputs and biomaterials, biotechnology, and bio-energies, especially local developments
v)	promote the incorporation of new technologies to improve productivity, with sustainability, paying attention to environmental care
vi)	encourage innovation and development of different crops, centred on regional economies

One of the fundamental aspects to support local biotechnology research was based on addressing the needs of domestic research groups (Lewi and Vicién, 2020). Concrete actions were implemented to support this, for example, the introduction of the biosafety greenhouses policy for local research. The guidelines for activities including regulated materials (GMOs) had been historically designed for companies requesting the import of seeds to carry out regulated trials in the country. But for local entities developing plant biotechnology, there were some aspects that required further clarity. One of these was the management of genetically modified (GM) plant material generated in laboratories, which had to be transferred to greenhouses under biosafety conditions. These transfers are carried out gradually, with plants grown *in vitro* requiring acclimatization in pots, first in growth chambers and then in greenhouses. The regulation at that time, which required about 6 months between the start of the application and obtaining an authorization, was not feasible. For this reason, a specific regulation was introduced to enable the transfer of plants developed in the laboratory to adjacent greenhouses.

Another policy action was introduced to address uncertainty regarding the regulatory pathway for newly developed local products. In this case, the Ministry of Agriculture provided a consultation window (“Does my product need to be regulated?”) in their website. The consultation comprises a series of questions for researchers that are then answered by the technical teams in the Innovation and Biotechnology Coordination, usually within a week. The answers offer advice on whether the product must be regulated by the current regulations, which regulations it should comply with and offering, if applicable, the possibility of a virtual meeting to delve deeper into any technical aspect^2^.

As discussed previously, supporting local research in biotechnology was a central focus for the 2020–2023 Argentine administration. As a result, local research in this sector (including plants, microorganisms, and animals), multiplied. To ensure consistent capacity building on biosafety guidelines and ensure compliance with regulations, the administration organised both virtual and in-person trainings (as far as pandemic restrictions allowed), to facilitate understanding and awareness of the regulatory framework and also providing specific consultations for research groups in public and private entities.

Another action was the improvement of the regulatory system and the promotion of new technologies. There is a greater need for new tools and ways of working that help both governments and regulators to better adapt to a changing world and to use regulation as an effective tool to stimulate and direct innovation (Armstrong et al., 2019).

Science is advancing at a rapid pace, and so far, regulation in Argentina has been keeping up with scientific and technological advances. This is in line with the “anticipatory approach” described by Armstrong et al. (2019), which emphasises flexibility, collaboration and innovation. In Argentina regulatory frameworks for different activities are continuously updated, improved, and strengthened, keeping pace with scientific innovations. In the context of GM plants: Argentina updated the evaluation of contained and confined activities and the environmental risk assessment guidelines (ERA), as both are requirements for commercial authorization (see Supplementary Material). These updates strengthened the procedures for evaluation and now include key concepts and methodologies in risk assessment, such as “problem formulation”, i.e., values to be protected, risk hypotheses, pathway to harm (García-Alonso, 2013), and data transportability (Vesprini et al., 2020; García Alonso et al., 2014). These guidelines allow fit-for-purpose risk assessments, considering that the breeding process mitigates unintended effects (Schnell et al., 2015), and avoid information redundancy by applying the concepts of familiarity and history of safe use (Capalbo et al., 2020).

In recent years, a wide variety of advances in biotechnology, molecular biology, and genome sequencing have emerged, these are commonly known by their English acronym, NBT (New Breeding Techniques). One of the best-known technologies within this group is “gene editing”, which allows a targeted and specific intervention in the genome.

In 2015, Argentina officially introduced the first NBT normative, applied only to plants. This resolution was the first in the world, providing specific guidelines for the potential regulatory pathways of products derived from new breeding techniques (NBTs), including gene editing. Years later, in 2019, specific normative for animals and microorganisms were also published, and the plant normative was updated.

As a result, Argentina has been a reference point for other countries in the region such as Chile, Brazil, Colombia, Paraguay, Honduras, Guatemala, and Uruguay, all of which have developed their own normative with similar viewpoints, consolidating the adoption of the same criteria in a large part of Latin America. Argentina has since actively participated in international forums and provided training to other countries.

After the experience gained in analysing applications for products derived from NBTs, in 2020 a unification, update, and simplification of the resolution for products derived from NBTs was performed, becoming official in 2021 (Resolution 21/21)^3^.

Argentine regulations are based on the definition of GMO used in the Cartagena Protocol, as “an organism that has been obtained through the application of modern biotechnology and that contains a new combination of genetic material.” In turn, the definition of “new combination of genetic material” is equivalent to that of “transgenic event” in Argentine regulations: “stable and joint insertion of one or more genes or sequences that are part of a defined genetic construct.” These definitions are accompanied by criteria that consider whether that product or improvement could have been obtained through conventional genetic improvement or could have been found in nature.

Cases are submitted individually for analysis at CONABIA through a Preliminary Consultation Instance (PCI). Consultations can include products that are still in the design stage or products already developed and in the commercialisation pathway.

In their response CONABIA confirms whether the product is a GMO or not. For products in the design stage, the response is drafted using the potential tense mode (i.e., the response concludes whether it would be a GMO) and the entities must re-consult when the product has been effectively obtained.

Since this system was implemented, an increased number of requests have been submitted by developers. In fact, an analysis of the PCI cases received showed that: (a) developers were better equipped to predict the costs and estimate the time for product development, even at the design stage. (b) NBT products that are not considered GMOs can enter the market faster than GMO products; (c) a greater variety of traits was introduced in different crop species, animals, and microorganisms, compared to GMO cases; (d) the dynamics and speed of innovation of products obtained by NBT is greater compared to GMOs (Whelan et al., 2020; Goberna et al., 2022).

### Regulatory diplomacy

Regulatory diplomacy is about forming and mobilising international networks that allow cooperation with other regulatory agencies worldwide (Warner and Pink, 2023). Argentina has actively engaged in regulatory diplomacy in the field of biotechnology which have provided opportunities for collaborative experimentation, iterative learning, and continuous improvement. Some examples of the main activities undertaken by the National Directorate of Bioeconomy between 2020 and 2023 are provided below.

The “Memorandum of Understanding between the Ministry of Economy of the Argentine Republic and the Ministry of Science, Technology and Innovation of the Federative Republic of Brazil for Cooperation on Biosafety of Modern Biotechnology Products”^4^ is an emblematic example of regulatory diplomacy, cooperation, and good practices. Regulators and decision-makers from both countries had been engaging for some time in informal discussions on regulatory oversight within the framework of the Biotechnology Commission of the SGT8 ″Agriculture” of MERCOSUR and the Working Group “Policies for Biotechnology” of the Southern Agricultural Council. But it was not until the end of 2020 and during 2021 that these discussions turned into concrete actions for defining common criteria and objective regarding agricultural biotechnology. The memorandum provides a framework for collaboration between the regulatory agencies of both countries, streamlining and facilitating the evaluation and approval of biotechnological products within the agricultural sector. The main objectives of the agreement are listed in Table 2.

**TABLE 2:** Main objectives of MOU Argentina- Brazil.

No	Objective	Refers to
1	to reduce costs and time	simplify regulatory processes for developers of biotechnological products, especially for small and medium-sized enterprises
2	to promote local and regional innovation	promote the development of new technologies in the agricultural sector, such as gene editing and biotechnology
3	to strengthen bilateral trade	facilitate the exchange of biotechnological products between both countries, avoiding unnecessary regulatory barriers and especially asynchronies in the authorization of products that potentially lead to disruptions in trade
4	to guarantee food safety	ensure that biotechnological products are safe for human and animal consumption, and that they do not have negative impacts on the environment

Under this agreement both countries conduct the joint evaluation of applications for authorization of biotechnological products. This introduces important efficiencies such as streamlining processes, reducing costs and facilitating synchronous approvals, which in turn facilitate bilateral trade. The key benefits of the agreement are: greater competitiveness, enabling developers from both countries to develop and commercialize biotechnological products more efficiently, increasing their competitiveness in the global market; sustainable development: the agreement contributes to the adoption of technologies that allow for increasing food production in a sustainable manner, reducing environmental impact; and strengthening the bilateral relationship: cooperation in the field of biotechnology deepening commercial and scientific ties between Argentina and Brazil. In summary, this agreement represents an important step towards the integration of the regulatory systems of both countries in the field of biotechnology, which fosters innovation, facilitates trade, and increases sustainable development in the region.

Argentina, Brazil, Paraguay, and Uruguay have taken a significant step towards working together in the development of science and technology applied to food production, with the creation of the International Network for Biosafety of Modern Biotechnology Products (ABRE-BIO). The network comprises institutions from each of the four countries that control and guarantee the biosafety of Genetically Modified Organisms (GMOs), gene editing and New Breeding Techniques (NBTs). In this context, biotechnology is viewed as a key element in modern agriculture, not only to meet the priority objective of producing healthier food, and in a more sustainable manner, as the world demands, but also to enable the generation of employment, development, and wellbeing in the rural areas of the countries involved.

The network was formalized through a multilateral memorandum of understanding signed on 23 August 2023, at the end of the Southern Agricultural Council (CAS) meeting and, formalized in June 2024. It is expected that more countries will join in the near future. It is expected that this network will allow for closer cooperation in innovation in the agricultural, agro-industrial, and agri-food sectors by four countries that make a key contribution to sustaining global food security. It will also promote the exchange of professionals and scientific information related to biosafety and risk assessment of GMOs and the technical evaluation of products derived from NBTs. It will also lead to reduction in cost and time in regulatory oversight. Additionally, it will provide opportunities for further harmonization. The countries in this network are also aligned in the aim of fostering local bio-developments, both public and private. This represents an example of deepening commercial relations, that do not compromise on safety and promote sustainable production systems and food security.

Argentina, through the National Directorate of Bioeconomy (DNB), promoted the creation of the Global South Bioinnovation Group (BIGSUR), a group of global countries in southern regions (Latin America, Africa, and Southeast Asia) that encourages the creation of an enabling environment for scientific research and innovation aimed at accelerating local solutions to food security, climate change, and biodiversity loss through active cooperation and joint actions.

BIGSUR held its first meeting on May 18 and 19, 2023, at the Secretariat of Agriculture, Livestock and Fisheries (SAGyP) in Buenos Aires, Argentina. The meeting was attended by regulatory representatives from 13 countries (Argentina, Brazil, Chile, Uruguay, Peru, Paraguay, Costa Rica, Colombia, Honduras, Nicaragua, Kenya, Nigeria, and Ghana), to discuss topics relating to agricultural biotechnology and gene editing and explore common positions regarding regulation and innovation. A second meeting co-organised by Argentina and Kenya took place August 23, 24, and 25 of the same year in Nairobi, Kenya. This time the meeting was attended by regulatory representatives from 19 countries (Argentina, Brazil, Uruguay, Paraguay, Colombia, Honduras, Kenya, Nigeria, Ghana, Burkina Faso, Ethiopia, Malawi, Mozambique, Rwanda, Zambia, Zimbabwe, Tanzania, Philippines, and Bangladesh).

The event aimed at consolidating the group of southern countries, creating a forum for harmonizing and coordinating regulations on innovative biotechnologies (such as gene editing), to share experiences and knowledge, and to align common messages to share in multilateral forums.

Within the framework of the event, the Argentine delegation from the DNB provided training to countries in the analysis of hypothetical cases of gene editing products, with the intention of enabling regulators and decision-makers in the countries to make science-based decisions to promote local developments and new biotechnological solutions to food security and climate change. The event aimed to improve participants’ understanding of how the regulatory approach affects the adoption of innovative technologies.

It is considered that genome editing techniques can add value to existing agricultural practices in a way that improves sustainability while respecting biodiversity and addressing climate change. Ongoing research with CRISPR/Cas9 and other techniques in Latin America, Africa, and Southeast Asia show great potential to contribute to the accelerated achievement of the Sustainable Development Goals (SDG).

Countries of the Latin America and the Caribbean (LAC) region, Africa, and Southeast Asia are currently at different stages of development of their regulatory frameworks for biotechnology. Understanding these complex and dynamic interactions is key for developing governance and investment strategies that are appropriate and acceptable for each region.

The Southern Agricultural Council (CAS) is another example of a regulatory forum that fosters dialogue, consultation, and coordination of actions at the ministerial and regional level on matters concerning the sustainable development of the agricultural, forestry, and fisheries sectors, animal and plant health, food safety, as well as international negotiations on trade in agricultural, fisheries, and forestry products. Composed of the Ministers of Agriculture, or their equivalents, of Argentina, Bolivia, Brazil, Chile, Paraguay, and Uruguay, it was established in 2003 through the signing of an agreement and ratified in 2005 on the VII Ordinary Meeting of the CAS. It is chaired by the Ministers of Agriculture of the member countries. The CAS has a network of regional technical groups that support and implement ministerial decisions. These specialized groups make up the Agricultural Policy Development Network (REDPA) and take part in different projects and actions according to the needs and priorities of the region. Its primary function is to define the topics and priorities of the regional agricultural and forestry agenda, as well as to articulate the development of the agreed actions.

As a sectoral forum for the analysis of the problems of sustainable development of the sector, CAS focuses particularly on evaluating development policies and programs, the progress of trade negotiations, and agreeing on positions for participation in multilateral, plurilateral, and bilateral forums with countries or blocs outside the region. Evaluating the sanitary and phytosanitary situation of the region and coordinating control and eradication actions for sanitary and phytosanitary problems, as well as coordinating positions in relation to the work carried out in international standardization forums are also leading activities of this forum. Working Group 5 (WG5) of the CAS - Public Policies on Biotechnology Working Group No. 5, called Public Policies on Biotechnology, meets regularly every year and has two declarations at the World Trade Organization (WTO) supporting new biotechnologies and best regulatory practices. In addition, in May 2023 WG5 published a document on biotechnology in the region aimed at providing information about the benefits of using biotechnology and its potential as a tool for agricultural production, biosafety in the environment, and food safety for both humans and animals^5^.

There are other instances where Argentina exercises regulatory diplomacy in negotiation forums for issues relevant to biotechnology and agriculture: the Convention on Biological Diversity, the Cartagena Protocol, the Organisation for Economic Co-operation and Development (OECD), etc., and other events where regulators from Argentina and BIGSUR countries meet to discuss and coordinate activities: such as the International Symposium of Biosafety Research, the Seed Association of the Americas Congress, Like Minded Group meetings, etc. These participations are essential to collaborate in the formulation of international agreements and regulations that directly impact the agricultural sector, as well as to prevent the advancement of binding issues that could affect the way biotechnology is produced and applied in the sector. It is also important to participate in events where the objective is to promote scientifically sound research that supports the evaluation of biosafety by improving communication between scientists who study plants, animals, and microorganisms with new characteristics produced through modern biotechnology. In summary, it is important to have a group of countries with similar ideas and common spaces where regulatory diplomacy can be exercised, a field that is closely linked to the intellectual and regulatory “battle” over agricultural technologies. These activities should be carried out always recognizing that agricultural production will need to be substantially increased to meet global food demand, understanding that innovative agricultural technologies must continue to play a critical role in addressing these challenges, and emphasizing that regulatory approaches must have a scientific basis.

Capacity building is another example where Argentina has been exercising regulatory diplomacy. Since 2014, Argentina has been recognized as a Reference Centre for Biosafety of Genetically Modified Organisms (GMOs) by FAO. This recognition has facilitated the country’s engagement with more than 20 countries^6^ strengthening their regulatory capacities and providing technical assistance with topics relating to the biosafety of modern biotechnology (Table 3). In 2023, the Agreement with FAO was renewed for the third time until2027^7^.

**TABLE 3:** Capacity building activities in the recent years.

Year	Country
2020/2023	Perú: project “Strengthening Capacities for Activities with New Breeding Techniques (NBT), including Gene Editing”, founding by the Argentine Fund for South-South and Triangular Cooperation “FO-AR”; Two missions in 2022 (Buenos Aires-Lima) and three missions in 2023 (Lima-Buenos Aires-Lima)
2019–2021	Cuba: Workshops for training Cuban technicians and officials in biotechnology and biosafety within the framework of the “FOAR Cooperation Project between Argentina and Cuba”
2020	Panama: Training course for Panamanian officials within the framework of a United Nations GEF Project, “Strengthening National Capacities for the Full Implementation of the Cartagena Protocol on Biosafety in Panama,” and with CONABIA as a global reference center for biotechnology training. November 2020
2021	Thailand: Webinar: Exchange of experiences in Agricultural Biotechnology in Argentina and Thailand. December 2021
2022	Egypt: Virtual training for officials and regulators from the Agricultural Research Center of Egypt, as part of a training requested by Egypt and organized by the Argentine Embassy in Egypt. Seminar on Modern Biotechnology for Agriculture and Agroindustry - Training provided to professionals from Egypt

With more than 30 years of experience in the evaluation, regulation, and promotion of agricultural biotechnology, and with the goal of facilitating technology transfer and bilateral trade between countries, Argentina has been one of the first countries to develop and apply biotechnology techniques to promote the development of primary production. These achievements also reflect FAO recommendations regarding their importance in improving food production, reducing production costs, benefiting the environment, and promoting developments that, in line with scientific advancements, allow to produce ever-increasing quantities of safe, harmless, and high-quality food.

In this regard, Argentina has collaborated with more than 20 countries, strengthening capacities and providing technical assistance on biosafety of modern biotechnology (Table 3).

The experience gained in Argentina demonstrates that, although there is heterogeneity in the progress and maturity of regulatory systems across countries, positions can be brought to closer alignment and a common regulatory language can be found, promoting the use of shared criteria. This was demonstrated by the discussions that took place during the BIGSUR workshops. Even when different countries were at different stages in their regulatory journey, the examples discussed revealed a number of similarities in criteria and conclusions. The application of sound scientific criteria and harmonized definitions facilitate this task. This was particularly evident at a workshop in Kenya in August 2023 where regulators from different countries met to analyse and discuss case studies of products derived from gene editing. Once the conclusions from these case studies were shared, it became clear that the criteria that the different countries had applied were similar and the conclusions reached were also similar. This exercise shows that, although countries may have different regulations or are at different stages of implementation, the application of scientific criteria and reasoning based on similar globally adopted definitions are fundamental. Another observation was that differences in terminology to the same processes and/or products in gene editing applied in different countries pose a challenge. To address this, the delegates agreed to adopt common terminology that would facilitate the differentiation between GMO products and gene edited products. Some of the definitions discussed related to the terminology associated with GMOs and NBTs. The proposal was that GMOs should be referred to as “GM” or “biotechnological or transgenic crops”, while those derived from NBTs -that are not considered GMOs-should be referred to as “non-GM” or “new product” or “variant”. Regarding the method used to develop the products, the proposal is to describe the methods to produce a GMO as genetic modification or transgenesis and for NBT products as “genetic intervention”. The steps leading to the commercial approval of these products are also different, while for GMOs, a pre-market risk assessment is conducted, for NBT products a technical evaluation is carried out to determine the regulatory status that leads to a conclusion or technical opinion. It is also important to emphasize that all products are regulated until they obtain their commercial authorization. In Argentina all crops require a variety registration that is overseen by National Seed Institute (INASE).

### Regulatory challenges (what lies ahead)

Until the publication of this review, Argentina’s NBT normative has been sufficient to resolve the regulatory status of the cases presented. Most of these cases referred to specific gene editing involving single base replacements or deletions of sections of a gene sequence to silence its expression. However, the advancement of scientific developments and the application of new technologies are leading the analysis towards more complex cases that could potentially challenge this normative.

Regulatory anticipation can maximize opportunities and mitigate the potential risks associated with emerging technologies (Armstrong, 2019). Anticipated applications of gene editing tools in the near future include multiple gene edits, gene duplication, allelic replacement, chromosomal rearrangements (inversions, translocations), polyploidization, and the utilization of gene editing to generate variability for genetic improvement.

In the broader context of agricultural biotechnology, the following challenges include: alternative protein production; cell-cultured food; molecular farming (production of molecules in alternative matrices); genetically engineered bio-inputs for agriculture and products impacting human and animal health, such as organs for xenotransplantation from genetically modified (GM) or gene-edited animals, GM insect vectors for disease transmission prevention, and GM insects for pest control. In this sense, international harmonization and cooperation would be crucial for addressing these challenges.

## Conclusion

The development of agricultural biotechnology and its regulatory framework has become a state policy in Argentina. Strengthening the institutional capacities for the regulatory oversight of biotechnology derived products has enabled the development of regulations based on scientific and technological advances. Notably, between 2020 and 2023 there was a special focus on providing support for innovation and development of local research.

These advances were enabled by building a highly qualified workforce with strong scientific backgrounds encouraged to follow the latest scientific developments and engaged with the international regulatory and scientific community.

While international forums propose to discuss and address regulatory harmonization, each country should develop its own regulations, considering its own sovereignty based on the values to be protected established in its constitutional principles, laws, and regulations. However, bilateral, regional, and multilateral dialogues are conducive to encourage the use of a common regulatory language and terminology, in which the definitions of the products subject to regulation and analysis are clear and agreed upon.

Successful international regulatory cooperation hinges on two critical conditions: fostering trust and facilitating knowledge exchange among regulatory bodies. These elements promote constructive dialogue, collaborative evaluation sharing, and the establishment of a trusted environment for discussing current and emerging challenges, particularly those posed by novel technologies. Such knowledge sharing streamlines communication and facilitates consensus building while respecting the sovereign decisions and perspectives of each nation. These dialogues should prioritize the wellbeing of stakeholders engaged in the scientific and technological advancement of each community, focusing on addressing specific local challenges. This approach stimulates interest in problem-solving, identifies necessary resources, and defines regulatory requirements for case-by-case assessments of agro-environmental biosafety and food suitability.

## Footnotes

1
https://www.argentina.gob.ar/sites/default/files/2022/07/nota_verbal_fao_sobre_conabia.pdf

2 Link to the consultation site: https://www.magyp.gob.ar/conabia/

3
https://magyp.gob.ar/sitio/areas/biotecnologia/conabia/_pdf/Resolution_N21-2021_3%20annexes.pdf

4
https://www.magyp.gob.ar/internacionales/mous-brasil.php

5
http://consejocas.org/wp-content/uploads/2023/07/Publicaci%C3%B3n-Evoluci%C3%B3n-del-uso-de-la-biotecnolog%C3%ADa-en-los-pa%C3%ADses-del-CAS.pdf

6
https://www.magyp.gob.ar/internacionales/pdf/Catalogo-de-Proyectos-FOAR-Area-Biotecnologia.pdf

7
https://www.argentina.gob.ar/sites/default/files/2022/07/ae_04–2023_4_1.pdf

## References

[B1] Armstrong H. Gorst C. Rae J. (2019). Renewing regulation – ‘Anticipatory regulation’ in an age of disruption. NESTA Lond. Available online at: https://media.nesta.org.uk/documents/working_model_for_anticipatory_regulation_0_TpDHt7z.pdf.

[B2] Capalbo D. M. F. Macdonald P. Fernandes P. M. B. Rubinstein C. Vicién C. (2020). Familiarity in the context of risk assessment of transgenic crops: focus on some countries in the Americas. Front. Bioeng. Biotechnol. 7, 463. 10.3389/fbioe.2019.00463 32047744 PMC6997124

[B3] García-Alonso M. (2013). Safety assessment of food and feed derived from GM crops: using problem formulation to ensure “fit for purpose” risk assessments. Collect. Biosaf. Rev. 8, 72–101. Available online at: https://foodsystems.org/wp-content/uploads/2021/02/GarciaAlonso.pdf.

[B4] García-Alonso M. Hendley P. Bigler F. Mayeregger E. Parker R. Rubinstein C. (2014). Transportability of confined field trial data for environmental risk assessment of genetically engineered plants: a conceptual framework. Transgenic Res. 23, 1025–1041. 10.1007/s11248-014-9785-0 24733670 PMC4204004

[B5] Goberna M. F. Whelan A. I. Godoy P. Lewi D. M. (2022). Genomic editing: the evolution in regulatory management accompanying scientific progress. Front. Bioeng. Biotechnol. 10, 835378. 10.3389/fbioe.2022.835378 35265604 PMC8900009

[B6] Lewi D. M. Vicién C. (2020). Argentina’s local crop biotechnology developments: why have they not reached the market yet? Front. Bioeng. Biotechnol. 8, 301. 10.3389/fbioe.2020.00301 32328485 PMC7160672

[B7] Schnell J. Steele M. Bean J. Neuspiel M. Girard C. Dormann N. (2015). A comparative analysis of insertional effects in genetically engineered plants: considerations for pre-market assessments. Transgenic Res. 24, 1–17. 10.1007/s11248-014-9843-7 25344849 PMC4274372

[B8] Vesprini F. Maggi A. I. López Olaciregui M. Módena N. A. (2020). Transportability of conclusions from confined field trials: a case study using the virus resistant transgenic bean developed in Brazil. Front. Bioeng. Biotechnol. 8, 815. 10.3389/fbioe.2020.00815 32850707 PMC7396523

[B9] Warner R. Pink G. (2023). “Navigating regulatory landscapes: four sights to advance regulatory practice and governance,” in Guest editorial, regulation policy and practice newsletter, APO. Edition 40. Available online at: https://anzsog.edu.au/news/navigating-regulatory-landscapes-four-sights-to-advance-regulatory-practice-and-governance/.

[B10] Whelan A. I. Gutti P. Lema M. A. (2020). Gene editing regulation and innovation economics. Front. Bioeng. Biotechnol. 8, 303. 10.3389/fbioe.2020.00303 32363186 PMC7181966

