# Supplementary material for: Experiences, learnings and perspectives in the regulation of agricultural biotechnology: the view from Argentina
Source: Front Bioeng Biotechnol. 2025 Jun 6;13:1600642. doi: 10.3389/fbioe.2025.1600642 (PMC12179121; doi:10.3389/fbioe.2025.1600642)
Supplement: Supplementary file 1 [file Table1.docx]

Table 1: Improved and updated policies for GMO plants

| Policy name | Policy number | Issues included | purpose | link |
| --- | --- | --- | --- | --- |
| ASSESSMENT OF CONTAINED OR CONFINED ACTIVITIES WITH REGULATED PLANT GMOs | Resolution No. 45/22 | activities in Biosafety Greenhouses for local developments, Biosafety Greenhouses for foreign developments, common greenhouses, and field activities, whether they be trials or seed or biomass production | provides all the necessary information to evaluate these types of activities in an orderly manner, focusing on the biosafety of the activities. | <https://servicios.infoleg.gob.ar/infolegInternet/anexos/365000-369999/367208/norma.htm> |
| CONTAINMENT CONDITIONS FOR TRIALS WITH REGULATED GM MATERIALS | Resolution No. 19/2021 | It refers to the conditions under which these activities must be carried out, including isolation distances from sexually compatible species and the number of years of control after harvest that the surface must remain unsown with the same crop, unless it is the same GMO. | Isolation distances and control after harvest | <https://servicios.infoleg.gob.ar/infolegInternet/anexos/345000-349999/346692/norma.htm> |
| RISK ASSESSMENT OF PLANT GMOs, AS A PRIOR REQUIREMENT FOR REQUESTING COMMERCIAL AUTHORIZATION | Resolution No. 32/2021 | to carry out an environmental risk assessment in the agroecosystems where the crop will be grown on a large scale once commercial authorization is obtained | This update strengthens the procedures for evaluation and includes the concepts of Problem Formulation, i.e. values to be protected, risk hypotheses, pathway to harm, data transportability (Vesprini et al., 2020; García Alonso et al., 2014), among others | <https://servicios.infoleg.gob.ar/infolegInternet/verNorma.do?id=347662> |
| Related to the regulation on environmental risk assessment (Resolution No. 32/2021) | CIRCULAR CIyB N°1 | Unintended Effects | It is known that genetic transformation can originate unintended effects. These are discarded during the selection process (Schnell et al., 2020). The absence of unintended effects that could pose a risk to the agroecosystem is confirmed in the agro-phenotypic characterization studies. Therefore, it is considered appropriate for the applicant to complete the environmental risk assessment, arguing about the unintended effects in relation to the risk of the GM plant on the agroecosystem, according to what is observed in the agro-phenotypic studies. | <https://www.magyp.gob.ar/sitio/areas/biotecnologia/conabia/_pdf/CIRCULAR_CIyB_N1_ENOI.pdf> |
| Related to the regulation on environmental risk assessment (Resolution No. 32/2021) | CIRCULAR CIyB N° 2 | focuses on expression products and crops with a history of safe use (HDUS) and familiarity. According to the regulation, the concept of familiarity is "the pre-existing scientific knowledge, experimental evidence, and accumulated regulatory experience on new expression products and/or on modified crops that can be taken into account in a risk assessment". | The gathering of documents, data, and existing literature can constitute supporting material and form the weight of the evidence for risk assessment. Likewise, the same regulation defines History of Safe Use (HDUS) as "a tradition of use, where scientific procedures or formal knowledge are not necessarily available or are limited. However, given the history that reports empirical evidence of use without adverse effects, it can be used as a weight of evidence to reach conclusions about the safety of new expression products, modified crops, and/or host crops". By applying these concepts, we aim to avoid the redundancy of information declared in the different environmental risk assessment applications (Capalbo et al., 2020). | <https://www.magyp.gob.ar/sitio/areas/biotecnologia/conabia/_pdf/CIRCULAR_CIyB_N2_HDUS.pdf> |
| Related to the regulation on environmental risk assessment (Resolution No. 32/2021) | CIRCULAR CIyB N°3 | addresses studies on the analysis of centesimal composition, toxicity, and allergenicity | Considering the precedent of “Circular N°1” and with the objective of conducting a new review of the evaluation criteria and information to be requested, it is considered that in the absence of risk hypotheses specifically related to the composition of the GM plant and its potential risk to the agroecosystem, the analysis of the centesimal composition does not provide new information and the absence of unintended effects could be determined through agro-phenotypic characterization studies. | <https://www.magyp.gob.ar/sitio/areas/biotecnologia/conabia/_pdf/CIRCULAR_CIyB_N3_ACCTA.pdf> |
